# How do aesthetics and tourist involvement influence cultural identity in heritage tourism? The mediating role of mental experience

**DOI:** 10.3389/fpsyg.2022.990030

**Published:** 2022-10-28

**Authors:** Wei Yang, Qiuxia Chen, Xiaoting Huang, Mei Xie, Qiuqi Guo

**Affiliations:** ^1^School of Management, Shandong University, Jinan, China; ^2^Yellow River National Strategic Research Institute, Shandong University, Jinan, China

**Keywords:** heritage tourism, aesthetics, tourist involvement, mental experience, cultural identity, ABC theory

## Abstract

As heritage is the precious treasure of human society, heritage also carries the genes of culture. It is of vital importance to effectively develop heritage tourism resources and explore the mechanisms that influence tourists’ cultural identity. This study has integrated the stimulus-organism-response (SOR) framework with the attitude-behavior-context (ABC) theory to construct a hypothetical model of heritage tourism aesthetics, tourist involvement, mental experience, and cultural identity so as to figure out their relationships. The questionnaires were collected to investigate the impact paths and mechanisms between heritage aesthetics, tourist involvement, mental experience, and cultural identity. The structural equation model was used to examine the relationship between heritage tourism aesthetics, tourist involvement, mental experience, and cultural identity. The main findings include: (1) the positive impact of aesthetics driving mental experience and cultural identity is significant; (2) the impact of tourist involvement on mental experience and cultural identity is also significant; (3) the impact of aesthetics on cultural identity is not significant, but mental experience mediates the relationship between aesthetics and cultural identity in heritage tourism. This study provides a new research framework and perspective for the aesthetics, tourist involvement, mental experience, and cultural identity of tourists in heritage tourism. This study also provides practical implications for government culture sectors to propagandize culture and for heritage destination managers to better manage heritage sites.

## Introduction

Culture is a unique phenomenon in human society. The material and spiritual wealth created by human beings in the process of transforming the natural environment belong to the category of culture. Heritage, appearing in Europe in the 1970s, includes a variety of material carriers such as local cultures, historical figures, folk art, and architectural styles which carries unique historical, cultural, and aesthetic values. And heritage is an effective way of cultural representation and an important carrier of cultural identity (Zhang R. et al., 2021; Xu et al., 2022; Zhang and Brown, 2022). Until 2022, China owns 56 World Cultural and Natural Heritage Sites recorded on UNESCO’s World Heritage List, making it one of the countries with the most complete types of World Heritage Sites in the world (Dai et al., 2022). Heritage tourism research began in the 1990s with a group of European scholars who were the first to explore the topics like heritage establishment and conservation, and gradually expanded by North American scholars who focused on natural heritage conservation and sustainable development in the context of protected areas and national park systems (Harvey, 2008; Harrison et al., 2020; Smith, 2020; Gandarillas and McCall, 2021; Santoro et al., 2021). Since China joined the UNESCO World Heritage Committee, heritage research has gradually flourished and the primary studies can be summarized in the following four themes: fundamental issues of heritage sites and sustainable development (Shen and Chou, 2021; Zhang and Lee, 2022; Zhou et al., 2022), heritage tourists (Fang et al., 2021; Gao et al., 2021; Xu et al., 2022), benefits of heritage tourism stakeholders and heritage communities (Chen, 2022; Zhang and Brown, 2022) and heritage tourism activities (Zhang and Lee, 2022). In addition, the Chinese government and heritage conservation departments have placed strategic importance on the preservation and inheritance of cultural heritage. This policy has guided and encouraged regional government departments to promote and protect cultural heritage by establishing conservation associations, renovating heritage sites, constructing museums, developing special towns, and organizing cultural heritage tourism activities (Yan, 2018, 2021; Zhang, 2020; Qiu and Zhang, 2021; Wang et al., 2021; Zhang S. et al., 2021).

Franklin (2003) argued that tourism is an important part of citizen’s life and tourists can experience national history and culture by participating in heritage tourism activities or rituals. Heritage tourism plays an important role in promoting national cultural identity and forming collective centripetal forces (Palmer, 1999; Zhang C. X. et al., 2018). Tourists’ behaviors and emotions during ritual activities in heritage tourism destinations, as well as performances and experiences, contribute to the construction of cultural identity (Grajzl et al., 2018; Zhang C. X. et al., 2019). For instance, visiting mausoleum sites creates a cultural identity for Taiwanese tourists to learn about their ancestral root culture (Wei et al., 2022). Gao et al. (2021) investigated Chinese tourists’ overall perceptions of national cultural heritage and found that tourists were interested in cultural heritage and heritage sites that transmit the country’s history and culture. Wei et al. (2022) conducted a 5-year field study at the Yellow Emperor’s Tomb in Shaanxi Province, China, and their study illustrated how heritage tourism enhanced the cultural identity of tourists through a specific social project.

However, based on reviewing past academic literature on heritage and culture, we found that, on the one hand, the majority is concerned with how heritage interprets cultural identity and the reasons behind it, especially the implicit function of cultural heritage to play an official and formal role in interpreting and enhancing cultural cohesion (Lowenthal, 1998; Bandyopadhyay et al., 2008; Zhou et al., 2022). However, few quantitative studies have been conducted on heritage tourism and cultural identity. On the other hand, heritage tourism presents culture in a way that tourists can easily accept and promotes the cultural understanding and identity of heritage tourists in the hedonistic context of tourism activities (Zhang L. et al., 2018; Gao et al., 2021). Heritage tourism plays a crucial part in promoting and disseminating culture (Liu et al., 2021), and it becomes a unique form and effective instrument for heritage tourists to receive historical and cultural education about the country in a subtle way. Moreover, tourists’ aesthetics of the environment is important to the relationship between heritage tourism and cultural identity (Zhang L. et al., 2018; Yang et al., 2022). Specifically, heritage tourism provides a unique cultural expression that achieves cultural awareness, understanding, and transmission through tourists’ gaze and aesthetics of heritage landscapes (Ren et al., 2021; Zhang R. et al., 2021). However, few studies have focused on the relationship in heritage tourism between heritage aesthetics and tourists’ mental experience, and cultural identity. Research also scarcely focuses on the relationship between aesthetics, tourist involvement, mental experience, and cultural identity, as well as the impact path and mechanisms of tourist involvement, mental experience, and cultural identity. Furthermore, at the level of industrial practice, while heritage tourism is booming worldwide, plenty of countries are dependent on heritage tourism as a communicative carrier to actively play an important role in promoting the cultural identity of their citizens (Barghi et al., 2017; Koya and Chowdhury, 2020). Based on previous analysis, the following research questions are proposed: in the modern heritage tourism context, how do tourists’ aesthetics of heritage sites and tourist involvement in heritage-related activities or rituals affect tourists’ cultural identity in the country where the heritage is located? What role does mental experience play as a mediating variable?

The main research objectives of this paper are: (1) to use structural equation modeling to explore the impact path of heritage tourism aesthetics and tourist involvement driving mental experience and cultural identity, respectively, from the perspective of environmental psychology and combing the SOR model with affect-behavior-context theory, and cultural identity in the World Cultural Heritage, the SanKong Scenic Area in China by conducting questionnaire research; (2) using mental experience as a mediator to investigate the impact of heritage tourism aesthetics and tourist involvement on cultural identity (cognitive identity, affective identity, and behavioral identity) and explore the mechanism of the impact.

Based on reviewing previous relevant academic literature, this study has the following primary theoretical contributions: first, ABC theory was originated from environmental psychology and the vast majority of the literature adopting this theory as a theoretical foundation pertained to the categories of pro-environmental behavior, green consumption, resource recycling, and public health (Yadav et al., 2019; Wu et al., 2021; Chopdar et al., 2022). There are barely studies applying ABC theory to the research of heritage tourism and cultural identity. Therefore, this paper combines the SOR model and ABC theory and applies them to the research of heritage tourism, expanding the extension and applicable situation of ABC theory. Second, most of the studies on the aesthetic aspects of heritage tourism focused on the fields of art and landscape architecture (Zhang R. et al., 2021; [Bibr B29][Bibr B104]), whose findings can be summarized as the value of heritage aesthetics, the connotations of heritage landscape aesthetics, and aesthetic deconstruction, but little attention has been paid to the impact of heritage tourism aesthetics on mental experience, tourist involvement, and cultural identity. Finally, in the test of mediating effects, this study found that mental experience plays a significant mediating role in the impact paths of both heritage tourism aesthetics and tourist involvement in cultural identity. This finding provides greater clarity on the internal impact mechanisms of heritage tourism on cultural identity. In addition, the findings of this study provide valuable practical insights for cultural departments in the country where heritage is located to carry out cultural promotion activities, and for heritage destination managers to enhance tourists’ aesthetic and mental experiences of heritage landscapes and promote tourists’ cultural understanding and identity behind heritage.

The paper are organized as follows: the first section introduces the current development and research progress of heritage tourism, and describes the research objectives, research perspectives, research questions, and theoretical contributions; the second section describes the literature review and hypothesis development; the third section elaborates and analyzes the theoretical foundation and proposes the research framework; the fourth section introduces the overview of the SanKong Senic Area, research methods, and data collection; the fifth section presents the data analysis and results. Finally, the last section introduces the findings of this study, discussions, theoretical and practical implications, and describes the limitations of this study and research prospects.

## Literature review, research framework, and hypothesis development

### Aesthetics

Aesthetics connotes sensory experience related to arts or concepts, such as form and expression, signal and image, beauty, taste, and feeling. Mathwick et al. (2001) defined aesthetics as a reaction to the symmetry, proportion, and unity of a physical object (e.g., environment and construction), a work of arts (e.g., music and poetry), or a performance. They deconstruct aesthetics into two dimensions: (1)noticeable visual components in a physical environment, such as visual appeal, hue, configuration, and resolution quality displayed by a picture; (2)the joy dimension of service. Therefore, all services deserve deliberate appreciation. As a focus of aesthetics, aesthetics based on vision is a structural way people observe and appreciate objects (Rancière, 2009; Trinh and Ryan, 2013). The cognitive process and mental mechanism for humans enjoying the natural landscape and man-made landscape as well as the impacts that the environment exerts on human behaviors have been researched. Berlyne (1972) discovered that when people appreciate a beautiful landscape, the visual processing depends on the physical, ecological, and integral propensity of the landscape to a great extent. For instance, aesthetic perception of landscape is significantly influenced by diversity, layers, and so on (Vanstockem et al., 2018). To state it simply, in modern aesthetics research, appreciating landscape in terms of vision depends on the integral exhibition effect of all components (Dramstad et al., 2006).

Scholars approve that aesthetics plays a crucial part in tourist destination image formation, marketing, and tourists’ mental experience (Kirillova et al., 2014). That’s to say, mental experience based on aesthetics is an essential content for Chinese tourists’ tourism experience. One of the most important motivations for Chinese tourists is to seek a mental experience of landscape aesthetics (Xu et al., 2013). The uniqueness of mental experience in heritage tourism literature is that most Chinese tourists prefer reciting and deconstructing ancient poetry which they saw at the cultural heritage site (Yu and Xu, 2016). Generally speaking, poetry can arouse tourists’ nostalgia and cultural pride so as to enhance their appreciation and understanding of heritage landscapes (Xu et al., 2013). That’s to say, aesthetic interpretation of Chinese ancient poetry, legends, myths, and arts can effectively promote tourist attractions appeal in order to boost tourists’ mental experience (Xu et al., 2013). Chinese classical gardens are the cultural heritage of China. Studying the spatial beauty of classical garden is of great significance to inherit conventional culture, conventional art, and concentional aesthetics (Guanglong and Qian, 2022; Zheng et al., 2022).

We can understand that aesthetic experience is a cognitive and processing process, among which aesthetic judgment is a cognitive part of this process. A tourist tries to have all senses actively taken part in the aesthetic processing of a tourist destination during appreciation (Kirillova et al., 2014).

### Tourist involvement

Tourism involvement has aroused researchers’ attention owing to its impacts on individual attitudes and decision-making, which has been considered a pivotal psychological construct in past 10 years (Taylor et al., 2018). This concept refers to the extent to which consumers invest when consuming products or services according to previous literature. Also, the tourist involvement refers to the state of tourists’ motivation and interest in the product or service (Gursoy and Gavcar, 2003). Despite the importance of tourist involvement in understanding consumer behavior, its lack of attention by tourism researchers due to the complexity of the concept and the inclusion of many segmented dimensions has led to it being largely neglected in research in the tourism literature (Jeong et al., 2019). In recent years, tourist involvement has been noticed by a few researchers and more and more scholars realize that tourist involvement is directly related to destination identity formation based on literature review. Heritage tourism can play a key role in providing a specific “ritualized environment” in which visitors can effectively engrave latent collective cultural genes and memories through participation in specific rituals and activities. At the same time, the experience of visitors’ participation in heritage tourism activities can be considered an important medium for promoting their cultural identity and consolidation.

Palmer (2005) indicated that tourist participation in specific cultural rituals (e.g., viewing, salutations, wreath-laying, worship, etc.) in heritage tourism destinations enables tourists to imagine and construct their own identity within the group, and can encourage folks to try to feel and consolidate individual connections to the nation’s history in their imagination by increasing tourist involvement. It promotes a different positioning of the individual within the broader context of cultural construction and symbolic embodiment of national identity. Chen’s (2022) research about Macau also confirmed the above sight.

### Mental experience in heritage tourism

Until now, there is no shortage of research on the mental experiences of tourists in tourism contexts. However, we lack a unified and clear perspective. We begin with the phenomenological view that the core of the mental experience in heritage tourism contexts lies in the visitor’s perception, understanding, and interactive relationship with heritage, which has been widely accepted in the literature. For example, some researchers deemed that in order to understand behaviors at a specific place, we need to explore the connection between humans and the visited place (Poria et al., 2006); discussion about the interaction between tourism background and the place; mental experience being created jointly (Herbert, 2001). That’s to say, tourists choose to actively interact with the external environment rather than passively receiving it. The result of visiting a site from Chronis (2005) is a “cultural narrative formed by the information provided in the exhibition is enriched and completed by the tourist’s historical knowledge and their personal struggle to follow this narrative through their imagination.” McIntosh and Prentice (1999) also proposed such an idea and their research figured out that through selective participation, tourists match the perceptual information gained with their knowledge and past experiences. Prentice and Andersen (2007) also found that tourists’ experience is unique in cultural heritage. It is essential for us to understand the process and details of tourist involvement as well as their relationship with heritage sites formed by the process and details so as to further understand mental experience in the cultural heritage tourism context. In other words, under the guidance of heritage tourism, tourists gaze at the characteristics and details of heritage sites, perceive, and understand the cohesive culture to form a unique mental experience. In brief, this study reasonably hypothesizes and generalizes that the mental experience is a unique experience generated by tourists through aesthetic and participatory behaviors in the tourism activities of cultural heritage, which is influenced by the aesthetic and interactive activities in the early stages and later enhance long-term cultural identity (Yang et al., 2022).

H1: Aesthetics in the heritage tourism context has a significantly positive impact on tourist involvement.

H2: Aesthetics in the heritage tourism context has a significantly positive impact on tourists’ mental experience.

H3: Tourist involvement has a significantly positive impact on tourists’ mental experience.

### Cultural identity

Cultural identity is deemed as the basic form of national identity as it is originated from the same historical cognition and shaped by culture. What’s more, cultural identity facilitates the identity and consolidation of an individual’s national identity. In an unusual tourism environment, the formation of the tourist’s own cultural identity relies on a long-term, deep cognition, and more stable emotion on the one hand, and on the other hand can be formed through a short-term cultural learning process. Several scholars highlight that a basic framework exists in individuals’ cultural cognition from childhood and they will incorporate continuous exposure to the group’s shared historical culture into that framework as they grow throughout their lives. Especially in the field of heritage tourism, tourism landscape (e.g., heritage, relics, exhibitions, specific rituals) uses a static, profound, and connotative historical culture, through an external form that is easy to understand for the public and a specific interactive experience to awaken the cultural genes deep inside the tourists (Zhang C. X. et al., 2019). Therefore, tourists’ relevant cultural memories in the tourism context are reproduced and consolidated due to the awakening of cultural genes, and the deepening of cultural memories further leads to tourists’ positive cultural identity, which completes the process from general exposure to superficial understanding and to deeper understanding (Zhang L. et al., 2018). More importantly, the formation of cultural identity is based on perception, understanding, and experience of culture (Kranz and Goedderz, 2020), while heritage tourism obviously offers tourists a suitable and efficient chance.

Heritage tourism provides a new atmosphere and a unique field for tourists’ cultural aesthetic and mental experience, and tourist involvement also promotes tourists’ cultural identity. A study of Jewish tourism heritage in culturally complex multi-ethnic areas found that heritage tourism promotes tourists’ cultural identity, even if this cultural identity is multifaceted and complex. Jan Packer et al.’s (2019) case study of the Australian War Memorial and Gallipoli Battlefield suggests that heritage tourism has an impact on tourists’ cultural identity. There is no doubt that visiting heritage sites can play an important role in enhancing the mental experience of tourists and strengthening cultural identity. Tourists engage in cultural ritual activities specific to cultural heritage destinations, a novel cultural expression catering to the market, which helps tourists understand the culture from superficial to deep (Zhang L. et al., 2018), and enhances their cultural perceptions and experiences. Though previous studies have emphasized the political role of purely visiting heritage tourism (e.g., visiting heritage, exhibitions, and war sites) on tourists’ identity (Gieling and Ong, 2016; Packer et al., 2019), they have neglected the important value of cultural heritage in terms of tourists’ cultural learning, experience, and identity in the context of dynamic tourism development (Zhang and Brown, 2022). Hence, the cultural identity of groups is proposed to be divided into three dimensions: cognitive, affective, and behavioral. This study attempts to clarify cultural identity in-depth in terms of these three dimensions of subdivision, whereby the following research hypothesis is proposed:

H4: Mental experience has a significantly positive impact on cognitive identity.

H5: Mental experience has a significantly positive impact on affective identity.

H6: Mental experience has a significantly positive impact on behavioral identity.

H7: Cognitive identity has a significantly positive impact on affective identity.

H8: Cognitive identity has a significantly positive impact on behavioral identity.

H9: Affective identity has a significantly positive impact on behavioral identity.

H10: Aesthetics has a significantly positive impact on cognitive identity.

H11: Tourist involvement has a significantly positive impact on affective identity.

## Stimuli-organism-response model and attitude-behavior-context theory

Mehrabian and Russell (1974) proposed the Stimulus-Organism-Response (R) theoretical analysis framework, referred to as the S-O-R analysis model. The framework explains that when an individual is exposed to an external stimulus (S), it affects the internal psychological state of the organism (O), which in turn leads to a response (R). Subsequent studies have shown that stimuli to the organism can alter people’s cognition and emotion, which can involuntarily result in changes in behavioral intentions. The stimulus-organism-response (SOR) analytical framework has been applied to various aspects of tourism literature like tourist decision-making behavior (Slama and Tashchian, 1987; Yadav et al., 2021; Yang et al., 2022), tourists’ consumption behavior (Ying et al., 2021; Huy et al., 2022), aesthetics of tourism landscape (Jani and Han, 2015), and tourist experiences (Ben Haobin et al., 2021; Shin and Jeong, 2022; Talwar et al., 2022), which contributes abundant knowledge to this literature. In association with the literature, the current study develops and tests an extended SOR model to predict potential tourists’ behavior in heritage tourism.

Attitude-behavior-context (ABC) theory originated from the research of environmental psychology (Stern and Oskamp, 1987). They proposed that individuals engage in pro-environmental behavior (PEB) as a result of a series of causal effects resulting from external and internal factors. Guagnano et al. (1995) further stated that internal situational attitudes (A) and external situational factors (C) and their interactions determine pro-environmental behavior (B). The theoretical perspective of environmental psychology argues that the causes of individual behavior are highly influenced by situational factors. However, attitudes could not effectively predict individual behavior if situational factors were not taken into account (Stern, 1999, 2000). In the ABC theoretical framework, “A” represents the organism’s attitude toward a specific behavior; “B” refers to the specific behavior of the organism; and “C” is the contextual factor (Guagnano et al., 1995). In subsequent studies, it has been argued that the “A” (attitude) in ABC theory refers to a person’s subjective factors, including beliefs, cultural orientations, values, and intentions, which tries to predict the intrinsic core of behavior (Ajzen, 2005; Zhang L. et al., 2018). The predictive role of subjective factors on individual behavior depends on contextual factors, such as contextual availability, interactive feedback, and social norms (Stern, 2000). ABC theory is now widely used in the study of environmental and individual behavior, such as the psychology and behavior of green consumption as well as resource recycling.

We argue that ABC theory is equally applicable to the study of tourist behavior in heritage contexts. According to ABC theory, when in a heritage tourism context, external landscapes such as heritage sites can be considered contextual (environmental) factors (Li et al., 2021; van Lanen et al., 2022). When individuals’ aesthetics toward heritage and engagement with cultural activities or rituals in heritage sites occur, cognitive and affective experiences resulting accordingly can be considered their attitudes toward heritage. Contextual (environmental) factors either hinder or facilitate individuals’ cultural identity (behavior) and explain the formation of cultural identity (behavior) together with individuals’ subjective attitude factors (Zhang L. et al., 2018). Therefore, when situational (environmental) factors are positive, cultural identity (behavior) is more likely to engender. Some researchers argued that the different impacts of attitudinal factors on individual behavior found in previous studies mainly ignored the role of environmental factors and tested only attitudinal factors (Stern, 1999, 2000; Goh and Balaji, 2016). However, Yadav et al. (2019) affirmed the importance of situational and psychological factors in determining travelers’ willingness to choose green hotels.

Attitude-behavior-context theory also has the following advantages as a theoretical foundation for the study: first, it is developed in the environmental psychology literature, therefore, adequately applied to explaining the behavior of tourists in heritage destinations. Second, because ABC theory combines individual subjective and situational (environmental) factors into the study of tourists’ cultural identity, it can help provide a more refined understanding of the formation mechanisms of cultural identity. Third, previous studies based on ABC theory showed that the impact path from attitude to behavior is significantly different in various contexts (environment) (Ertz et al., 2016; Zhang L. et al., 2018). However, most previous studies have focused on natural environment protection and green consumption (Yadav et al., 2019; Wu et al., 2021). However, few studies have been conducted in cultural heritage contexts. Hence, the study can bridge the gap in the literature and enrich the theoretical outreach and applicability of ABC theory.

**FIGURE 1:**
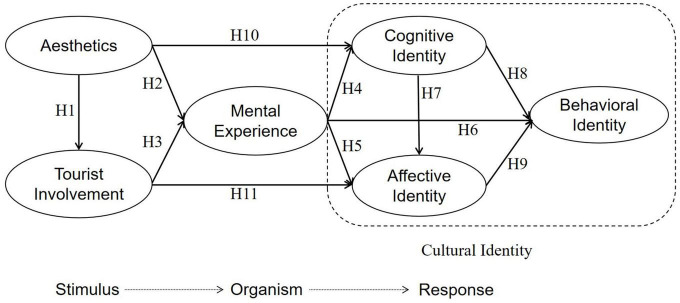
Tourists’ cultural identity framework in heritage tourism.

Therefore, we integrate the SOR model with the ABC theory to propose the research framework shown in Figure 1.

## Research design

### An overview of Qufu Area

**FIGURE 2:**
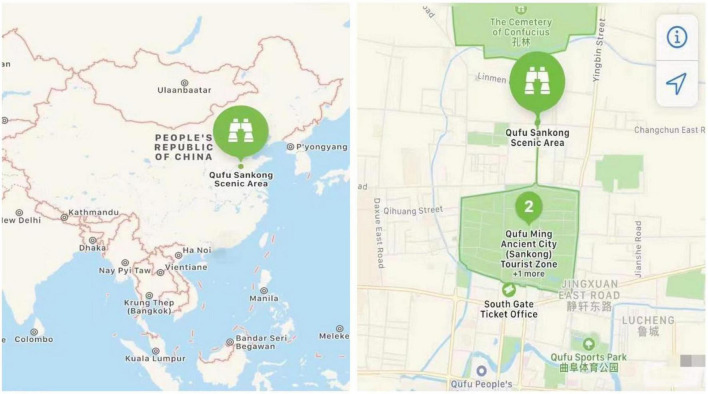
Qufu Sankong Scenic Area.

With a history of more than five thousand years, Qufu is the legendary birthplace of many Chinese sages, such as Confucius and Mencius. The most renowned attractions are the Temple of Confucius (Kong Miao), the Cemetery of Confucius (Kong Lin), and the Kong Family Mansion (Kong Fu), together now called the SanKong shown in Figure 2. The SanKong Scenic Area were listed in the World Cultural Heritage by UNESCO in 1994, along with a comment that said: “The Qufu complex of monuments has retained its outstanding artistic and historic character due to the devotion of successive Chinese emperors over more than 2,000 years.” Located in the center of Qufu City, Shandong Province, China, the Temple of Confucius was built in the 17th year of the Duke of Liao (478 BC) as an ancestral temple to Confucius, the famous ancient Chinese thinker and educator. It is one of the three major ancient architectural complexes in China, together with the Forbidden City in Beijing and the Summer Palace in Chengde. And one of the four major literary temples in China, together with the Fuzi Temple in Nanjing, the Confucius Temple in Beijing, and the Temple of Literature in Jilin. Confucius was named by UNESCO as one of the top ten cultural figures in the world. Confucius founded the doctrine of Confucianism, the core ideas of which are benevolence, righteousness, propriety, wisdom, and faith. After more than 2,000 years of inheritance and development, Confucianism has gradually become the orthodox culture of China. And it has deeply influenced countries in East and Southeast Asia even the whole world. There are altar and pavilion relics, memorial buildings, temples and tombs, and stone inscriptions and towers in SanKong Scenic Area. As the largest, best-preserved, and oldest surviving Confucius temple in China, the architecture of Confucius Temple in Qufu is not only a historical witness to the development and growth of Confucianism in the nation, but an important carrier for the comprehensive embodiment of Chinese classical architecture and architectural culture aesthetics.

**FIGURE 3:**
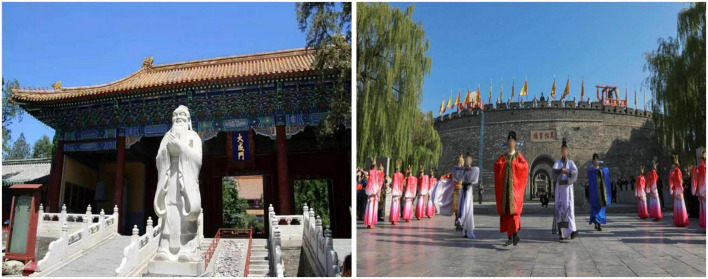
Heritage tourism in Qufu Sankong Scenic spot.

The opening ceremony of the Ming Dynasty City is held at the SanKong Scenic Area, which includes the auspicious morning bell, a dance to welcome guests, and guests entering the city (Figure 3). In addition, the SanKong Scenic Area will hold a ceremony to accept students on behalf of Confucius, in which tourists and their kids can participate to take Confucius as their teacher so as to know better about the ancient ceremony. Tourists can definitely feel the charm of national culture, through various forms of activities to participate in, resulting in a unique mental experience.

### Research method

The measurement items are shown in Table 1. The aesthetics (AE) scale used was based on Kah et al. (2010) and Boley et al. (2011). To match this research context and provide more concise items, we adapted original scales to this study and we got five items finally. This new scale tested its validity and reliability with Exploratory factor analysis (EFA). Mental experience (ME) was measured with five items originating from Wei et al. (2019). Tourist involvement (TI) was measured using six items by adopting existing literature (Afonso et al., 2018; Zatori et al., 2018). Cognitive identity (CI) has three items derived from Johnson et al. (2012) and Knez and Eliasson (2017). Affective identity (AI) has four items by adopting previous literature (Johnson et al., 2012; Knez and Eliasson, 2017; McGowan et al., 2017). Behavioral identity (BI) was measured with three items of word of mouth, re-visit, and recommendation adapted from Zhao et al. (2022).

**TABLE 1:** Measurement items.

Construct	Items	Descriptions of items	Supporting literatures
Aesthetic (AE)	AE1	I choose this cultural heritage site for its attractiveness.	Kah et al., 2010; Boley et al., 2011
	AE2	When I see this cultural heritage site, my full attention is absorbed by it.	
	AE3	Scenic culture creates a sense of awe within me.	
	AE4	When I plan to travel, I would like to travel to this cultural heritage site for its intrinsic value and exterior beauty.	
Tourist involvement (TI)	TI1	There are many activities to participate and interact with.	Zhang and Lu, 2010; Fan et al., 2019; Jeong and Kim, 2021; Setiawan et al., 2021
	TI2	I am involved in the event or ritual of the attraction.	
	TI3	The activities here catch my attention.	
	TI4	I feel fun from visiting the various attractions here.	
	TI5	I gain a different kind of travel experience by participating in the activities.	
	TI6	I gain a sense of involvement and experience in participation.	
Mental experience (ME)	ME1	I feel very happy during this trip.	Wei et al., 2019
	ME2	I am indulged in activities during this travel experience.	
	ME3	I did something meaningful on this trip.	
	ME4	I met myself on this trip.	
	ME5	This travel experience made me particularly excited.	
Cognitive identity (CI)	CI1	There exists a link between this cultural heritage site and my daily life.	Knez and Eliasson, 2017
	CI2	I can reflect on the memories attached to this cultural heritage site.	
	CI3	Thoughts about this cultural heritage site are part of me.	
Affective identity (AI)	AI1	I miss the traditional rituals experienced in this cultural heritage site.	Knez and Eliasson, 2017
	AI2	I am proud of this cultural heritage site.	
	AI3	I have strong ties to this cultural heritage site.	
	AI4	I know these traditional rituals quite well now.	
Behavior identity (BI)	BI1	I will recommend this cultural heritage site to others.	Zhao et al., 2022
	BI2	I tend to visit this cultural heritage site again in the future.	
	BI3	I will say positive things about this cultural heritage site to others.	

The questionnaire was designed based on the literature review and heritage tourism site’s specific situation, as follows. A 5-point Likert scale (“1” stands for strongly disagree and “5” means strongly agree) was adopted. The questionnaire was divided into seven parts. The first six parts consisted of six latent variables, namely, heritage tourism aesthetics in SanKong Scenic Area, Qufu city, tourist involvement, mental experience, cognitive identity, affective identity, and behavioral identity, and the last part was demographics with four items-gender, age, education background, and occupational background. We collected 545 questionnaires totally, deleting invalid and incomplete data, and finally, we got 460 qualified questionnaires.

Generally speaking, the sample size should be at least 10 times the sum of the number of all items in the questionnaire (Bentler and Chou, 1987). If stable and convincing results are expected, the valid sample size for this collection should be more than 300 (Loehlin, 1992; Tandon et al., 2020). Therefore, this study has met the requirement of sample size for one normative research.

### Data collection

The research was conducted at the tourist attraction- the SanKong Scenic Area in Qufu, Shandong, China, mainly targeting Chinese tourists. The research questionnaire was collected from August to September and November 2021 at the core scenic areas of the Temple of Confucius, the Cemetery of Confucius, and the Kong Family Mansion, respectively. Questionnaires were distributed to visitors at the rest areas of attractions such as Dacheng Hall, Dacheng Gate, Xingtan, Thirteen Stele Pavilion, and Kuiwen Pavilion, as well as in the recreation areas. Most visitors were free and willing to cooperate in completing the survey so a systematic random sampling method was adopted for the selection of samples. Before completing the questionnaire, we confirmed that the participants were tourists who visited the SanKong Scenic Area for pleasure, not for working-related reasons, and explained the study process to each participant. The researcher promised to keep their personal information and privacy confidential, and the results of the questionnaire would only be used for scientific research and not for commercial purposes. Rewards have been prepared to give participants after completing the research to compensate for taking up their leisure time.

## Data analysis

### Descriptive statistics analysis

The demographic characteristics of cultural heritage tourists have four dimensions, including gender, age, education background, and occupational background. The majority are male tourists (56.5%); most tourists are aged 26–40 (60.4%), followed by 18–25 (19.3%). Tourists possess bachelor’s degrees including college (86.3%), indicating that cultural heritage tourists have higher cultural cognitive ability. The major occupations are business managers and employees (49.0%), followed by students (27.6%). Given the analysis of the demographic characteristics, we conclude that the samples are nicely representative.

### Reliability and validity test

#### Reliability test

We use SPSS 27.0 to test Cronbach’s alpha of each construct so as to measure the internal consistency of questionnaires. The Cronbach’s alpha for the whole questionnaire was 0.906, and the Cronbach’s coefficient for each of the constructs was greater than 0.8. That’s to say, corresponding indicator of each item was >0.7, indicating that the questionnaire had relatively good internal consistency reliability (Cronbach, 1951). The result has been displayed in Table 2.

**TABLE 2:** Results of confirmatory factor analysis.

Construct	Items	Cronbach’s alpha
Aesthetic	4	0.820
Tourist involvement	6	0.888
Mental experience	5	0.854
Cognitive identity	3	0.842
Affective identity	4	0.855
Behavior identity	3	0.835
Overall	25	0.908

#### Composite reliability and convergent validity

Composite reliability (CR) and convergent validity were tested. The composite reliability and squared multiple correlation (SMC) could further test the stability, internal consistency, and reliability of each variable. The results of the reliability and convergent validity tests for the above six variables are shown in Table 3. All factors were close to or higher than the loading coefficient of 0.7 (*p* < 0.001), with no cross-loading, indicating that there is statistical significance. Moreover, the SMC values of the most factors were greater than 0.5 and CR values were greater than 0.7, further indicating that the reliability of each variable is good. In terms of convergent validity, the average extracted variance (AVE) of each factor was greater than 0.5, indicating that each item had a good convergent validity. The factor loadings (Std.) of six latent variables are all above the standardized factor loadings of 0.6 (as shown in Table 3), indicating that the six latent variables can represent each item ideally. Additionally, the composite reliability (CR) is above 0.8, which is higher than the standard of 0.7 (Bagozzi and Yi, 1988; Centobelli et al., 2020). And the average variance extracted (AVE) is higher than the standard of 0.5 (Hair et al., 2019), which indicates that the actual results of composite reliability and convergent validity are good.

**TABLE 3:** Results of composite reliability and convergent validity.

Latent variables	Items	Significance estimation of parameters				Factor loadings	Item reliability	Composite reliability	Convergent validity
		Unstd.	S.E.	*t*-value	*P*	Std.	SMC	CR	AVE
Aesthetic	AE4	1				0.712	0.507	0.822	0.538
	AE3	1.011	0.075	13.438	***	0.708	0.501		
	AE2	1.188	0.079	15.031	***	0.834	0.696		
	AE1	0.931	0.073	12.796	***	0.670	0.449		
Tourist involvement	TI6	1				0.713	0.508	0.890	0.577
	TI5	1.132	0.067	16.848	***	0.836	0.699		
	TI4	0.858	0.062	13.907	***	0.686	0.471		
	TI3	0.792	0.056	14.149	***	0.698	0.487		
	TI2	1.208	0.072	16.759	***	0.831	0.691		
	TI1	0.916	0.058	15.720	***	0.777	0.604		
Mental experience	ME5	1				0.644	0.415	0.863	0.560
	ME4	1.288	0.101	12.772	***	0.708	0.501		
	ME3	2.121	0.152	13.934	***	0.794	0.630		
	ME2	1.692	0.118	14.355	***	0.830	0.689		
	ME1	1.386	0.104	13.373	***	0.751	0.564		
Cognitive identity	CI1	1				0.788	0.621	0.844	0.644
	CI2	1.040	0.064	16.270	***	0.767	0.588		
	CI3	1.131	0.065	17.435	***	0.850	0.723		
Affective identity	AI4	1				0.720	0.518	0.856	0.600
	AI3	1.010	0.072	14.076	***	0.704	0.496		
	AI2	1.295	0.083	15.678	***	0.788	0.621		
	AI1	1.471	0.087	16.901	***	0.875	0.766		
Behavior identity	BI1	1				0.723	0.523	0.840	0.638
	BI2	1.451	0.090	16.109	***	0.844	0.712		
	BI3	1.263	0.079	15.900	***	0.824	0.679		

***p < 0.001; S.E., standard error (the same below).

As shown in Table 4, the value of the square root of AVE for each latent variable is higher than the value of the correlation coefficient between that latent variable and the other latent variables (Fornell and Larcker, 1981), which indicates that the discriminant validity of aesthetics, tourist involvement, mental experience, cognitive identity, affective identity, and behavioral identity is desirable.

**TABLE 4:** Discriminant validity.

	AVE	AE	TI	ME	CI	AI	BI
AE	0.538	**0.733**					
TI	0.577	0.419	**0.760**				
ME	0.560	0.474	0.488	**0.748**			
CI	0.644	0.216	0.204	0.405	**0.802**		
AI	0.600	0.216	0.343	0.359	0.324	**0.776**	
BI	0.638	0.22	0.257	0.413	0.57	0.497	**0.799**

The bold diagonal value represents the square root of AVE value. Off diagonal values represents coefficients between latent variables. AE, aesthetics; TI, tourist involvement; ME, mental experience; CI, cognitive identity; AI, affective identity; BI, behavioral identity (the same below).

### Model fitness test

Combining the SOR framework and the heritage tourism context, we use the Maximum Likelihood method through AMOS23.0 to perform the fitness test of the model. In the structural model (as shown in Table 5), CMIN/DF is 1.203 < 3. RMSEA is 0.049 < 0.08. GFI is 0.914 > 0.9. NFI is 0.909 > 0.9. CFI is 0.950 > 0.9. IFI is 0.950 > 0.9. TLI is 0.943 > 0.9. Consequently, the overall model fits well.

**TABLE 5:** Results of model fitness.

Fitness indicator	*CMIN/DF*	*RMSEA*	*GFI*	*NFI*	*CFI*	*IFI*	*TLI*
Recommended value	<3	<0.08	>0.9	>0.9	>0.9	>0.9	>0.9
Real/actual value	2.095	0.049	0.916	0.909	0.950	0.950	0.943

### Structural equation model analysis

#### Path analysis of overall structural model

Next, the hypotheses are tested by structural equation modeling: (1) to test the impact of aesthetics on tourist involvement and mental experience; (2) to test the impact of tourist involvement on mental experience; (3) to test the impact of mental experience on cognitive identity, affective identity, and behavioral identity; (4) to test the impact of cognitive identity on affective identity and behavioral identity; (5) to test the impact of affective identity on behavioral identity; (6) to test the impact of aesthetics on cognitive identity, affective identity, and behavioral identity. The results of the empirical analysis of the structural equation model are shown in Table 6. The main effects of the structural model are analyzed based on the fitness test of the structural model.

**TABLE 6:** Research hypothesis.

Hypothesis paths	Estimate	S.E.	*t*	*P*	Test results
H1: Aesthetic → Tourist involvement	0.516	0.071	7.285	***	Support
H2: Aesthetic → Mental experience	0.247	0.044	5.586	***	Support
H3: Tourist involvement → Mental experience	0.216	0.035	6.116	***	Support
H4: Mental experience → Cognitive identity	0.503	0.085	5.948	***	Support
H5: Mental experience → Affective identity	0.186	0.072	2.587	0.010 (**)	Support
H6: Mental experience → Behavioral identity	0.165	0.065	2.529	0.011 (**)	Support
H7: Cognitive identity → Affective identity	0.184	0.049	3.728	***	Support
H8: Cognitive identity → Behavioral identity	0.401	0.055	7.280	***	Support
H9: Affective identity → Behavioral identity	0.353	0.060	5.920	***	Support
H10: Aesthetic → Cognitive identity	0.031	0.060	0.513	0.608	Not supported
H11: Tourist involvement → Affective identity	0.149	0.040	3.709	***	Support

***p < 0.001, **p < 0.05.

**FIGURE 4:**
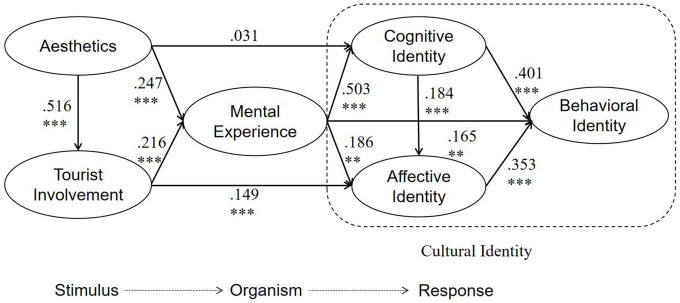
Result of the SEM. The symbol ** is significant at the 0.01 level and the symbol *** is significant at the 0.001 level.

According to the results, the impact of aesthetics on tourist involvement is significant (β = 0.516, *p* < 0.001), supporting H1. The impact of aesthetics on mental experience is significant (β = 0.247, *p* < 0.001), supporting H2; impact of tourist involvement on mental experience is significant (β = 0.216, *p* < 0.001), supporting H3. The impact of mental experience on cognitive identity is significant (β = 0.503, *p* < 0.001), supporting H4. The impact of mental experience on affective identity is significant (β = 0.186, *p* < 0.001), supporting H5. The impact of mental experience on behavioral identity is significant (β = 0.165, *p* < 0.05), in support of H6. The effect of cognitive identity on affective identity is significant (β = 0.184, *p* < 0.001), therefore in support of H7. The effect of cognitive identity on behavioral identity is significant (β = 0.353, *p* < 0.001), therefore in support of H8. The effect of affective identity on behavioral identity is significant (β = 0.353, *p* < 0.001), in support of H9. The effect of aesthetics on cognitive identity is not significant (β = 0.031, *p* = 0.608), rejecting H10. The effect of tourist involvement on affective identity is significant (β = 0.149, *p* < 0.001), in support of H11. All results above based on structural equation modeling analysis can be categorized into different paths. Each relational path and its standardized path coefficient are shown in Figure 4.

#### Mediating effect analysis

Mediating effects are tested by Bootstrapping function of AMOS 23.0. In the overall effect from aesthetics to cognitive identity, Bias-Corrected 95% CI from the lower bound value of 0.069 to the upper bound value of 0.32 does not contain 0, and Percentile 95% CI from the lower bound value of 0.069 to the upper bound value of 0.316 also does not contain 0. Thus, the total effect of heritage tourism aesthetics on cognitive identity exists. In the direct effect, bias-corrected 95% CI from the lower bound value of -0.171 to the upper bound value of 0.107 contains 0 while bias-corrected 95% CI from the lower bound value of -0.166 to the upper bound value of 0.122 contains 0. Therefore, the direct effect does not exist in both cases (as in Table 7). Mental experience plays a partially mediating role between heritage tourism aesthetics and cognitive identity (Hayes and Preacher, 2014). In the total effect of tourist involvement on affective identity, 0 was not included from the lower bound value of 0.122 to the upper bound value of 0.459 for bias-corrected 95% CI and also from the lower bound value of 0.12 to the upper bound value of 0.457 for Percentile 95% CI, indicating the presence of a total effect of tourist involvement on affective identity. In the case of direct effects, the bias-corrected 95% CI does not contain 0 from the lower bound value of 0.024 to the upper bound value of 0.363, and the Percentile 95% CI does not contain 0 from the lower bound value of 0.024 to the upper bound value of 0.363, indicating the existence of direct effects in both cases. Mental experience partially mediates the relationship between tourist involvement and affective identity. Other path effects are displayed jointly, and the tested hypothesis model is shown in Figure 4.

**TABLE 7:** Results of the mediating effect.

Path effect	Point estimation	SE	*t*	Bias-corrected 95%CI			Percentile 95%CI		
				Lower Upper		*P*	Lower Upper		*P*
AE → TI → CI → BI	0.022	0.007	3.143	0.013	0.040	0.000	0.012	0.038	0.000
AE → TI → AI → BI	0.007	0.004	1.750	0.002	0.017	0.013	0.001	0.016	0.024
AE → TI → BI	0.018	0.010	1.800	0.003	0.043	0.018	0.002	0.042	0.024
AE → TI → CI → AI → BI	0.004	0.001	4.000	0.002	0.008	0.000	0.001	0.007	0.001
AE → ME → CI → BI	0.050	0.015	3.333	0.028	0.087	0.000	0.027	0.085	0.000
AE → ME → CI → BI	0.008	0.003	2.667	0.004	0.017	0.000	0.003	0.016	0.001
AE → ME → BI	0.041	0.019	2.158	0.007	0.083	0.018	0.006	0.081	0.024
AE → ME → AI → BI	0.016	0.009	1.778	0.003	0.039	0.015	0.002	0.036	0.024
AE → CI → BI	0.012	0.026	0.462	−0.038	0.064	0.598	−0.039	0.063	0.620
AE → CI → AI → BI	0.002	0.004	0.500	−0.005	0.012	0.528	−0.006	0.011	0.621
TI → ME → CI → BI	0.044	0.012	3.667	0.024	0.074	0.000	0.023	0.072	0.000
TI → ME → AI → BI	0.014	0.007	2.000	0.003	0.032	0.013	0.002	0.030	0.024
TI → ME → BI	0.036	0.019	1.895	0.005	0.079	0.021	0.004	0.078	0.024
TI → ME → AI → BI	0.007	0.002	3.500	0.003	0.014	0.000	0.003	0.013	0.001
TI → AI → BI	0.053	0.020	2.650	0.021	0.105	0.001	0.018	0.098	0.002

## Conclusion and discussion

### Conclusion

This study conducted a survey of heritage tourists in one of China’s famous world heritage sites, the Sankong Scenic Area in Qufu City, Shandong Province, China. This study integrates the S-O-R theoretical framework and the ABC theory in the context of heritage tourism to construct a logically analytical framework for aesthetics in heritage’ tourism, tourist involvement, mental experience, and cultural identity, and empirically analyze the relationships and impact paths among the variables. The main findings: (1) the aesthetics of heritage tourism drives the formation of tourists cultural identity (cognitive identity, affective identity, and behavioral identity); (2) previous studies have not examined the impact of heritage tourism aesthetics on tourist involvement, and this study empirically tests that aesthetics has a significantly positive impact on tourist involvement, indicating that heritage tourism aesthetics can significantly enhance tourists’ engagement behavior; (3) heritage tourism aesthetics cannot directly influence cognitive identity, and mental experience plays a mediating role between aesthetics and cognitive identity. As a result, mental experience is an essential variable that ultimately influences the formation of tourists’ cognitive identity. Tourists perceive, memorize, and associate heritage tourism resources and landscapes to generate mental imagery and affective experiences in heritage tourism contexts, which in turn influences the formation of tourists’ cognitive identity; (4) mental experience partially mediates the relationship between tourists’ involvement and affective identity.

### Discussion

#### Theoretical implication

Based on the review of past academic literature, the main theoretical contributions of this paper are in the following: first, most of the past academic literature on heritage and culture analyzed the hidden meaning and relationship between heritage and culture (Zhang R. et al., 2021; Xu et al., 2022), or the impact of different storytelling on tourists’ cultural perceptions (Sigley, 2021), and heritage’s contribution to the affective and cultural identity of local residents (Zhang and Brown, 2022). The analytical methods were mainly from disciplinary backgrounds such as history, archeology, sociology, and anthropology. Thus, there is a paucity of quantitative research addressing the impact of heritage tourism and cultural identity. This study collected 460 questionnaires from visitors to World Heritage sites and used structural equation modeling to analyze them, boldly utilizing quantitative analysis methods to conduct the study. Therefore, this study makes up for the lack of quantitative studies on heritage tourism and cultural identity research.

Second, ABC theory was developed in environmental psychology. The vast majority of previous academic literature adopted the theory as a theoretical foundation were from the domains of pro-environmental behavior, green consumption, resource recycling, and public health (Yadav et al., 2019; Wu et al., 2021; Chopdar et al., 2022). There are almost no studies using ABC theory in the field of heritage tourism and cultural identity. Therefore, this paper combines and applies the SOR model and ABC theory to the study of heritage tourism, expanding the outreach and application context of ABC theory.

Furthermore, most of the studies had been conducted on the aesthetic aspects of human perceptions of heritage landscapes, which are primarily on art and landscape gardening (Zhang R. et al., 2021; Guanglong and Qian, 2022; Zheng et al., 2022), and conclusion include the value of heritage aesthetics, the connotation of heritage landscape aesthetics, and aesthetic deconstruction, but little attention has been paid to the impact of heritage tourism aesthetics on mental experience, tourist involvement, and cultural identity.

Finally, in the test of mediating effects, this study found that mental experience plays a significant mediating role in the impact paths of both heritage tourism aesthetics and tourist involvement in cultural identity. This finding demonstrates the internal impact mechanism of heritage tourism on cultural identity.

#### Management implication

Aesthetics and tourist involvement have a significantly positive impact on tourists’ mental experience. Thus, it is crucial to preserve, restore, and exhibit heritage tourism resources well. On the one hand, heritage resources can be made alive and more aesthetically diverse with the help of sound, light, and electricity. On the other hand, tourism destination management can provide more interesting tourism activities that fit the theme of the scenic spot and allow tourists to participate so as to attract tourists to visit, contact, and learn. Tourists can have more opportunities to appreciate the beauty of heritage resources from various dimensions. In addition, tourist involvement can also influence affective identity through tourists’ mental experience. Tourist destinations should take immersive tourism performance and other immersive tourism programs into account to further encourage tourist involvement. Furthermore, heritage tourism destinations should train tourism practitioners regularly to guarantee a quality service so as to help visitors better cognitively process information about heritage resources and thus promote tourists’ aesthetics. Or it can be realized by adding easy-to-understand explanatory notices for tourists.

The mental experience of tourists in heritage tourism plays a mediating role in the impact that tourists’ aesthetics and tourist involvement have on cultural identity, indicating that mental experience is an important variable that profoundly affects the cultural identity of tourists. Therefore, managers of heritage tourism destinations should pay attention to tourists’ mental experience, highlighting the aesthetic presentation of heritage resources. In order to attract tourists, heritage site management should pay attention to designing varied and interesting activities to encourage tourists to participate, and then enhance tourists’ awareness of the intangible values of history and culture through tangible heritage products (Dimache et al., 2017). It is quite efficient to awaken tourists’ collective memory and psychological emotions, deepen their construction and identification with the common culture and finally turn them into behavioral reality (Zhang C. X. et al., 2018).

### Limitations and future prospects

Certainly, this study has limitations. First, many moderating effects that affect the impact path between mental experience and cultural identity have not been tested, such as the types of tourist destinations. Since there are many different nations and countries in the world, the collision and intermingling of different cultures is also existing. Thus, future research can explore whether there are differences in heritage aesthetics and tourist involvement from different ethnic groups in their cultural identity and constructed pathways (Zhang C. X. et al., 2019). Second, this paper only uses questionnaires so the data source is a bit single. And samples only include Chinese tourists. Future research can contribute to the reliability of the findings by enriching the sample of tourists from different geographical regions like Europe and the Middle East (Huang and Liu, 2018).

## Data availability statement

The original contributions presented in this study are included in the article/supplementary material, further inquiries can be directed to the corresponding author.

## Ethics statement

Ethical review and approval was not required for the study on human participants in accordance with the local legislation and institutional requirements. The patients/participants provided their written informed consent to participate in this study.

## Author contributions

WY: method, analysis and interpretation of data, conclusion, implication, and drafting of manuscript. WY, QC, and QG: acquisition of data. QC: preparing figures and tables and visualization. XH: critical revision, study conception, and design. MX: acquisition of data and visualization. All authors contributed to the article and approved the submitted version.
